# Evaluation of Disk Halo Size and Identification of Correlated Factors in Myopic Adults

**DOI:** 10.3389/fmed.2022.743543

**Published:** 2022-01-28

**Authors:** Wuxiao Zhao, Jing Zhao, Tian Han, Meng Li, Jifang Wang, Xingtao Zhou

**Affiliations:** ^1^Eye Institute and Department of Ophthalmology, Eye & ENT Hospital, Fudan University, Shanghai, China; ^2^NHC Key Laboratory of Myopia (Fudan University), Key Laboratory of Myopia, Chinese Academy of Medical Sciences, Shanghai, China; ^3^Shanghai Research Center of Ophthalmology and Optometry, Shanghai, China; ^4^Shanghai Engineering Research Center of Laser and Autostereoscopic 3D for Vision Care (20DZ2255000), Shanghai, China

**Keywords:** halo size, higher-order aberrations, pupillometry, contrast sensitivity, myopia

## Abstract

This study aimed to evaluate glare source-induced disk halo size and assess its correlation with higher-order aberrations (HOAs), pupillometry findings, and contrast sensitivity in myopic adults (aged 23.8 ± 4.4 years). In this cross-sectional study, 150 eyes of 150 patients were assessed. All patients underwent routine ophthalmic examinations, wavefront aberrometry, halo size measurement, dynamic pupillometry, and contrast sensitivity tests. Spearman's correlation analysis and independent sample *t*-tests were performed for data analysis. The mean halo radius was 82.5 ± 21.8 and 236.7 ± 52.2 arc min at 5 and 1 cd/m^2^ luminance levels, respectively. The values were inversely correlated with internal spherical aberration (SA) (*r* = −0.175, *p* = 0.032 and *r* = −0.241, *p* = 0.003, respectively), but not correlated with spherical equivalent (SE, both *p* > 0.05). Positive correlations were observed between halo radius and pupil size, contraction amplitude, and dilation speed during pupillary light reflex. Halo radii at 5 and 1 cd/m^2^ luminance levels were not significantly correlated with the area under the log contrast sensitivity function (*r* = −0.093, *p* = 0.258 and *r* = −0.149, *p* = 0.069, respectively). The mean halo radius was not clinically different between myopic and healthy eyes at 5 cd/m^2^ luminance level and did not differ significantly between the high and low-to-moderate myopia at 5 and 1 cd/m^2^ luminance levels (all *p* > 0.05). According to a stepwise linear regression model, the internal SA had a negative effect on the halo radius under low photpic condition; the average pupil diameter, internal SA and corneal HOAs played a large role in determining the halo radius under mesopic condition.

## Introduction

Intraocular scattering is induced when strong light crosses the media in the eye. As a result, veiling light may be produced over the retina, thereby deteriorating the retinal image and contrast sensitivity. Therefore, glare (1) and halo (2) can occur. They have received much attention from investigators because they have been shown to reduce the safety of night driving (3) and impair general vision of patients who have undergone cataract or refractive surgeries (4–6). However, how glare impairs contrast sensitivity is relatively unknown.

Several studies based on subjective questionnaires have investigated the relationship between patient-reported symptoms and higher-order aberrations (HOAs) and showed that HOAs might be correlated with visual symptom scores (index for the severity of symptoms obtained from the questionnaire) in patients after laser *in situ* keratomileusis (7, 8). Accumulating evidence has revealed that HOAs and glare are relevant (9, 10). Visual symptoms, such as glare source-induced halo, have been measured quantitatively using a vision monitor device, and the halo size has been established in a normal, non-clinical population (11). A recent study shows that the halo size of low photopic luminance (5 cd/m^2^) is around 157.4 ± 56.7 arc min from a large number of myopic patients and claims that the size of halo is correlated with both spherical equivalent (SE) and minimum pupil size when pupillary light reflex is mediated (12). However, the halo size was not measured with an optimal correction and was clinically larger than that of healthy eyes of subjects (with uncorrected visual acuity ≥ 20/25 and similar age), and the measured halo size was 88.4 ± 22.1 arcmin using the same device at 5 cd/m^2^ (Metrovision, Pérenchies, France) (11). Therefore, whether there is a clinical difference in the size of halo (≥30 arc min, which is the interval scale for halo size measurement and it was also statistically significant) between myopic and healthy eyes and between high and low-to-moderate myopic eyes needs to be further investigated.

In addition, both haloes and HOAs can affect visual functions. The intraocular scattering affects the light distribution of the retinal image, and the size of the halo provides an estimate for an angle domain of >1°. However, HOAs mainly degrade the central peak of the point spread function (PSF), causing light to spread ~0.1°. It would be interesting to examine whether there would be a relationship between halo size and HOAs in myopic patients. We examined the halo size in individuals who exhibit different degrees of myopia under low photopic and mesopic luminance levels. We also investigated how disk halo correlated with other factors (e.g., HOAs, pupillometry, and area under the log contrast sensitivity function [AULCSF]).

## Materials and Methods

### Participants

In this cross-sectional study, 150 eyes of 150 myopic patients (aged 23.8 ± 4.4 years, 52 males and 98 females) who underwent preoperative routine examinations at the Eye, Ear, Nose & Throat (EENT) Hospital of Fudan University between July and October 2020 were recruited. The mean spherical equivalent (SE) of the group was −6.01 ± 2.01 diopters (range: −1.00 to −10.38 diopters). This study adhered to the principles of the Declaration of Helsinki, and the study protocol was approved by the EENT Hospital ethics committee (Registration number: ChiCTR1800017594). All patients provided an informed consent before the study.

The inclusion criteria were: myopic patients or those who had myopic astigmatism, aged 18–33 years, and corrected distance visual acuity of ≥20/20; those who had stopped using contact lens for at least 1 week, and those who had stopped using lens that were permeable to rigid gas or orthokeratology lens for at least 3 months before the study; an intact refractive status (annual variation of myopic power ≤ 0.50 diopters); ④ and no disturbance in night vision. The exclusion criteria were: ① patients with any refractive media opacity; ② eyes with active inflammation, trauma or surgical history, or systemic diseases; and ③ the usage of drugs that affect the movement of the iris.

All patients underwent routine ophthalmic examinations, including slit-lamp microscopy, uncorrected distance visual acuity, intraocular pressure, axial length, corneal topography (Pentacam HR, Oculus Optikgeräte, Germany), subjective refraction, and wavefront aberration (OPD-Scan, Nidek, Japan), halo size measurement, pupillometry, contrast sensitivity test (MonPack One, Metrovision, France), and fundoscopy. Patients were divided into high (SE range: −6.25 to −10.38 diopters) and low-to-moderate myopia (SE range: −1.00 to −6.00 diopters) groups for comparisons (13).

### Measurements

#### Wavefront Aberration

Wavefront aberrations of the human eye are defined as deviations from the ideal wavefront. OPD-Scan III (Nidek, Gamagori, Japan), which is based on automatic retinoscopy and features 2,520 aberrometry measurements, was used to measure wavefront aberrations of all included eyes (14). This device is designed to provide ocular, corneal, and internal HOAs across various pupil diameters. In this study, we collected data by measuring vertical Zernike coefficients, including ocular, corneal, and internal HOAs, for a 6-mm pupil.

#### Halo Size

Halo size was measured using a method similar to that reported by Puell et al. (11). A glare-inducing light source with a brightness of 200,000 cd/m^2^ was installed on both sides of the monitor (Metrovision, Pérenchies, France). The light source positioned to the right of the patient was used for the measurement of the right eye, and that positioned to the left of the patient was used for the measurement of the left eye. In this study, the luminance of the optotypes (arranged in three radial lines of letters presenting from the periphery toward the light source) was set to 5 and 1 cd/m^2^. The viewing distance was 2.5 m, after 5 min of dark adaptation, the light source was turned on followed by the optotypes presented on the monitor, patients with best-corrected spectacles were asked to read optotypes from the opposite side of the light source. When the observers told us that they were unable to recognize the shown letters, we recorded the visual angles, which were marked as halo radius and calculated in arc min (Supplementary Figure 1). In addition, the light source and vision monitor would be turned off simultaneously. One eye from each patient was randomly selected for the measurements.

#### Pupillometry

Pupillometry was performed by a skilled technician in the same location as the other tests. To reduce the influence of circadian rhythm on pupillometry, all measurements were completed during the same time period (09:00–11:00). The procedure was similar to that described in previous studies (12, 15, 16). The patient's head was placed on the chin rest, and the patient was asked to gaze at the center of the monitor. The MonPack One^®^ system (Metrovision, Pérenchies, France) with near-infrared illumination (880 nm) and a high-resolution infrared camera was used and allowed automated real-time image processing of pupil parameters with a measurement sensitivity of 0.1 mm.

**Figure 1:**
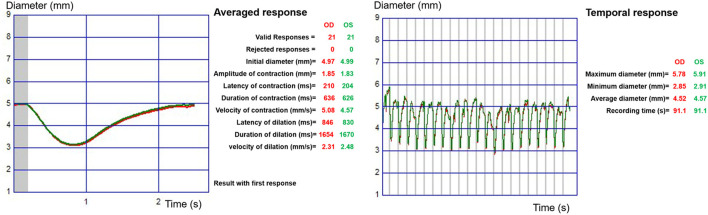
Dynamic pupillometry output data determined via the automatic quantitative pupillary vision monitor system (MonPack One, Metrovision, France). At the left, an averaged response is shown, and at the right, a temporal response is shown.

After the observers underwent 15 min of dark adaptation, their pupillary light reflex was able to be mediated with white-light flashes (stimulation on time 200 ms, stimulation off time 3,300 ms, total luminance 100 cd/m^2^). We recorded the response using an infrared camera. The valid response was measured at least 10 times in a 90-s period. We calculated it as the dynamic pupillary response of each eye. Twelve parameters, including average response parameters (initial pupil diameter, contraction amplitude, contraction latency, contraction time, contraction speed, dilation latency, dilation time, and dilation speed) and temporal response parameters (maximum pupil diameter, minimum pupil diameter, and average pupil diameter) were recorded for our data analysis (see Figure 1).

#### Contrast Sensitivity

As described by Zhao et al. (17), to measure the contrast sensitivity of the observers, we showed a sinusoidal grating at various spatial frequencies (cycles per degree (cpd) under a visual angle of 1°), such as of 0.5, 1.1, 2.2, 3.4, 7.1, and 14.6 cpd. Contrast sensitivity (luminance: 80 cd/m^2^) was measured using the MonPack One^®^ system at a distance of 2 m. Contrast sensitivity was measured monocularly and recorded sequentially (Supplementary Figure 2).

Contrast = (L_max_ – L_min_)/(L_max_ + L_min_) where L_max_ is the maximum luminance of the grating, and L_min_ is the minimum luminance of the grating.

Measures of contrast sensitivity are given in decibels (dB) and are in a logarithmic scale. *C* (dB) = −10 × log (Contrast). The AULCSF was determined by calculating the area under curve (i.e., integrals) of best-fitted curve of contrast sensitivity as a function of spatial frequency (from 0.5 cpd to 14.6 cpd).

### Statistical Analysis

SPSS 24 statistical software (IBM Corp., Armonk, NY, USA) was used for data analysis. Quantitative data are described as mean ± standard deviation in this study. The Kolmogorov–Smirnov test was used to assess whether the data were normally distributed. Spearman correlation was used to analyze the linear correlation between halo radius and refraction, axial length, HOAs, pupillometry parameters, and contrast sensitivity. A stepwise regression analysis was performed to evaluate the contributions of the variables. An independent sample *t*-test was used to compare the differences between the high myopia and low-to-moderate myopia groups. Bonferroni correction was used to correct the *p*-values for multiple comparisons. *P*-values <0.05 were deemed as statistically significant.

## Results

A total of 150 subjects (52 men and 98 women) with myopia and myopic astigmatism were enrolled in this study. Of the 150 subjects considered, 75 exhibited high myopia, and 75 had low-to-moderate myopia. Demographic characteristics and the refractive status of the participants are shown in Table 1. With best optical correction, the mean halo radius was 82.53 ± 22.84 and 236.73 ± 52.21 arc min at luminance levels of 5 and 1 cd/m^2^, respectively.

**Table 1:** Demographic and refractive data.

	**All eyes** **(***n =*** 150)**	**High myopia** **(***n =*** 75)**	**Low-to-moderate myopia (n= 75)**
Age (years)	23.84 ± 4.38 (18–33)	24.20 ± 4.19 (18–33)	23.48 ± 4.57 (18–33)
Sphere (D)	−5.58 ± 2.00 (−10.00 to −0.50)	−7.21 ± 1.00 (−10.00 to −6.25)	−3.95 ± 1.27 (−5.75 to −0.50)
Astigmatism (D)	−0.86 ± 0.58 (−3.25 to 0.00)	−0.87 ± 0.61 (−3.25 to 0.00)	−0.85 ± 0.56 (−2.50 to 0.00)
Spherical equivalent (D)	−6.01 ± 2.01 (−10.38 to −1.00)	−7.65 ± 1.04 (−10.38 to −6.25)	−4.38 ± 1.27 (−6.00 to −1.00)
Axial length (mm)	26.06 ± 1.04 (23.94 to 29.21)	26.59 ± 0.92 (23.39 to 29.21)	25.52 ± 0.86 (23.94 to 27.56)
CDVA (logMAR)	−0.01 ± 0.03 (−0.10 to 0.00)	−0.01 ± 0.03 (−0.10 to 0.00)	−0.01 ± 0.04 (−0.10 to 0.00)

*Values are represented as mean ± standard deviation*.

*CDVA, corrected distance visual acuity; logMAR, logarithm of the minimum angle of resolution*.

### HOAs

Table 2 shows the ocular, corneal, and internal HOA data based on a 6-mm pupil diameter. Independent sample *t*-tests showed that no significant difference was detected between the high and low-to-moderate myopia groups (α_new_ = 0.0042). Spearman correlation analysis showed that the halo radius was not significantly related to ocular and corneal HOAs (including total HOAs, coma, trefoil, and spherical aberration [SA]) at luminance levels of 5 and 1 cd/m^2^ (Supplementary Table 1), but was significantly correlated with internal SA (*r* = −0.175, *p* = 0.032 and *r* = −0.241, *p* = 0.003, respectively).

**Table 2:** Participant OPD-scan data (zone: 6 mm, *n* = 150).

**Parameters**		**High myopia**	**Low-to-moderate myopia**	***t***	***P***
		**(***n =*** 75)**	**(***n =*** 75)**		
Zernike/Ocular	HOAs (μm)	0.35 ± 0.12	0.38 ± 0.13	−1.438	0.152
	Coma (μm)	0.17 ± 0.10	0.17 ± 0.10	−0.488	0.626
	Trefoil (μm)	0.20 ± 0.12	0.23 ± 0.13	−1.428	0.156
	SA (μm)	0.12 ± 0.08	0.12 ± 0.08	0.102	0.919
Zernike/Cornea	HOAs (μm)	0.40 ± 0.11	0.43 ± 0.15	−1.437	0.153
	Coma (μm)	0.21 ± 0.11	0.21 ± 0.13	0.059	0.953
	Trefoil (μm)	0.14 ± 0.09	0.19 ± 0.10	−2.789	0.006
	SA (μm)	0.26 ± 0.07	0.27 ± 0.09	−0.855	0.394
Zernike/Internal	HOAs (μm)	0.38 ± 0.13	0.40 ± 0.16	−0.987	0.325
	Coma (μm)	0.20 ± 0.10	0.20 ± 0.10	−0.025	0.98
	Trefoil (μm)	0.17 ± 0.10	0.18 ± 0.13	−0.898	0.371
	SA (μm)	0.20 ± 0.12	0.21 ± 0.10	−0.605	0.546

*HOAs, higher-order aberrations; SA, spherical aberration*.

*Bonferroni Correction (α_new_ = 0.0042)*.

**Figure 2:**
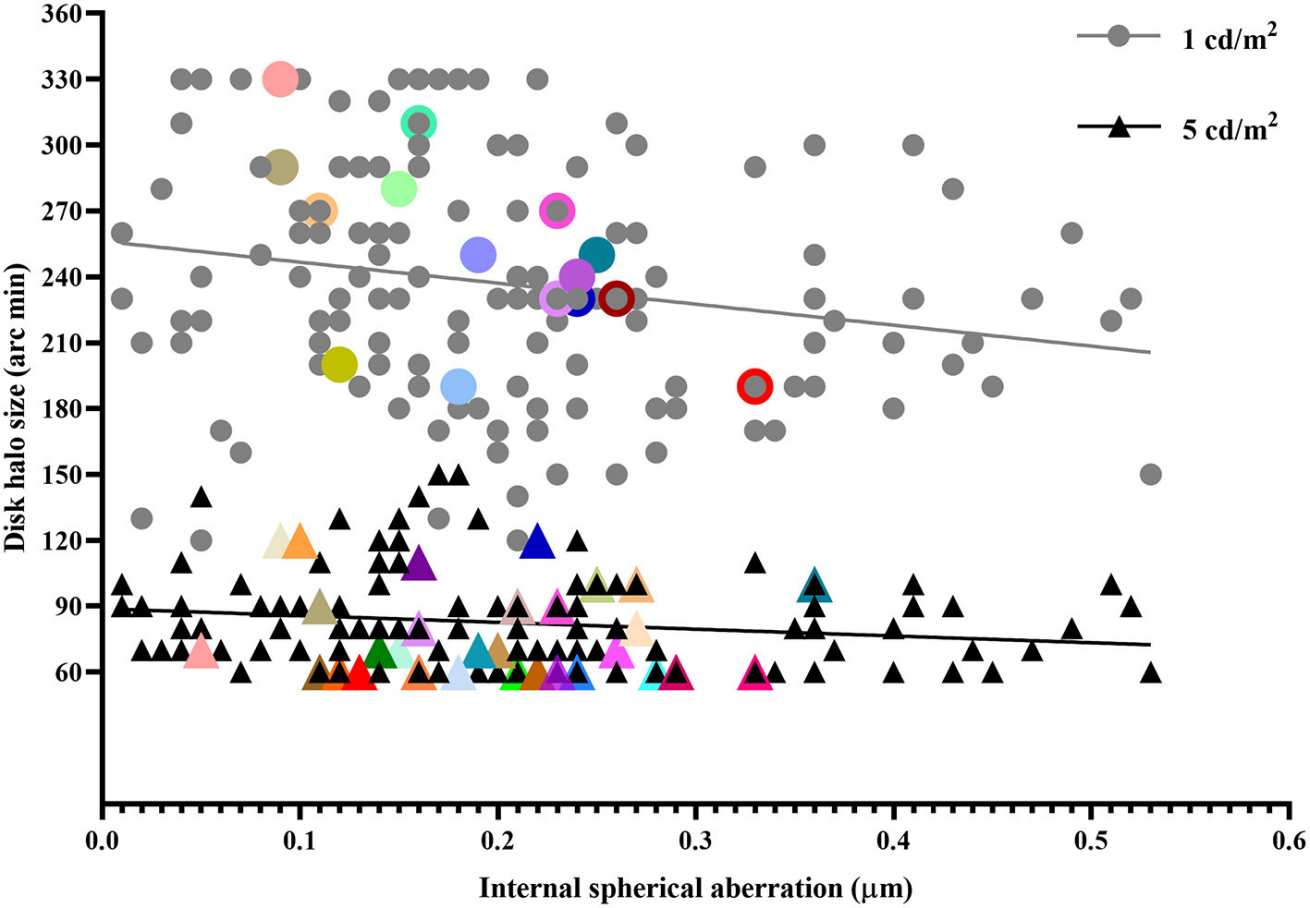
Disk halo size as a function of internal spherical aberration in patients with myopia and myopic astigmatism (*n* = 150). The overlapped dots were characterized by bigger size and different color.

Linear regression equations between the halo radius and internal SA were: *Y*_halo_ = −31*X*_iSA_ + 88.9 (*F* = 3.95, *R*^2^ = 0.026, *p* = 0.05) and *Y*_halo_ = −95.6*X*_iSA_ + 256 (*F* = 6.68, *R*^2^ = 0.043, *p* = 0.01) at a luminance level of 5 and 1 cd/m^2^, respectively (Figure 2). No correlation was noted between the halo size and other parameters.

### SE and Intergroup Comparisons

For mean halo radius and pupil size comparisons, an independent sample t-test revealed no significant difference between the high and low-to-moderate myopia groups, except with respect to SE (*t* = −17.225, *p* = 0.000, Table 3). Moreover, Spearman correlation analysis showed that a significant relationship was not found between SE and halo radii at luminance levels of 5 and 1 cd/m^2^ (*r* = 0.026, *p* = 0.748 and *r* = 0.082, *p* = 0.032, respectively).

**Table 3:** Halo radius and dynamic pupil values in different myopic groups.

	**High myopia**	**Low-to-moderate myopia**	***t***	***P***
	**(*n =* 75)**	**(*n =* 75)**		
Halo radius, 5 cd/m^2^ (arc min)	80.80 ± 20.45	84.27 ± 23.14	−0.972	0.333
Halo radius, 1 cd/m^2^ (arc min)	232.80 ± 51.77	240.67 ± 52.69	−0.922	0.358
Age (years)	24.20 ± 4.19	23.48 ± 4.57	1.006	0.316
Spherical equivalent (diopter)	−7.65 ± 1.04	−4.38 ± 1.27	−17.225	0.000
Initial pupil diameter (mm)	4.83 ± 0.59	4.89 ± 0.56	−0.675	0.501
Maximum pupil diameter (mm)	5.30 ± 0.69	5.34 ± 0.56	−0.394	0.694
Minimum pupil diameter (mm)	2.88 ± 0.39	2.89 ± 0.41	−0.234	0.815
Average pupil diameter (mm)	4.30 ± 0.49	4.36 ± 0.49	−0.740	0.460

### Dynamic Pupillometry

Dynamic pupillometry demonstrated that the mean response parameters, including initial pupil diameter, contraction amplitude, contraction latency, duration of contraction, contraction speed, dilation latency, duration of dilation, and dilation speed, were 4.86 ± 0.57 mm, 1.82 ± 0.24 mm, 2,230.28 ± 38.73 ms, 622.91 ± 57.62 ms, 5.65 ± 0.72 mm/s, 853.19 ± 49.46 ms, 1,638.46 ± 50.95 ms, and 2.07 ± 0.26 mm/s, respectively (Supplementary Table 2). For temporal response parameters, the maximum, minimum, and average pupil diameter were 5.32 ± 0.63, 2.88 ± 0.40, and 4.33 ± 0.49 mm, respectively. Spearman correlation analysis indicated that the average response parameters (including initial pupil diameter and dilation speed) and temporal response parameters (including maximum, minimum, and average pupil diameter) were positively correlated with halo radii at 5 and 1 cd/m^2^, whereas contraction amplitude was positively correlated with halo radius at 1 cd/m^2^ (*r* = 0.167, *p* = 0.041; Table 4).

**Table 4:** Correlations between halo radius and dynamic pupillometry in the study population (*n* = 150).

**Parameters**		**Disk halo size (arc min)**	
		**5 cd/m^2^ (r, P)**	**1 cd/m^2^ (r, P)**
Averaged response	Initial PD, mm	0.259, 0.001	0.260, 0.001
	Amplitude of contraction, mm	0.153, 0.062	0.167, 0.041
	Latency of contraction, ms	−0.154, 0.060	−0.134, 0.103
	Duration of contraction, ms	0.130, 0.113	0.121, 0.140
	Velocity of contraction, mm/ms	0.072, 0.380	0.051, 0.539
	Latency of dilation, ms	0.049, 0.554	−0.061, 0.461
	Duration of dilation, ms	−0.053, 0.518	−0.084, 0.307
	Velocity of dilation, mm/ms	0.207, 0.011	0.171, 0.037
Temporal response	Maximum PD, mm	0.313, 0.000	0.297, 0.000
	Minimum PD, mm	0.294, 0.000	0.271, 0.001
	Average PD, mm	0.297, 0.000	0.291, 0.000

*PD, pupil diameter*.

### Contrast Sensitivity

At spatial frequencies of 0.5, 1.1, 2.2, 3.4, 7.1, and 14.6 cpd, contrast sensitivity was 16.43 ± 2.02, 20.42 ± 1.74, 21.80 ± 1.70, 21.57 ± 1.88, 19.57 ± 2.34, and 11.86 ± 2.34 dB, respectively. Moreover, the calculated AULCSF was 28.44 ± 2.32 dB. Spearman correlation analysis showed that halo radii at 5 and 1 cd/m^2^ were not significantly correlated with AULCSF (*r* = −0.093, *p* = 0.258 and *r* = −0.149, *p* = 0.069, respectively).

### Multivariate Stepwise Regression Analysis

At 5 cd/m^2^ luminance level, the internal SA had a negative effect on the halo radius [halo = 88.965 – (31.586 × internal SA)] according to a stepwise linear regression model (*F* = 4.085, adjusted *R*^2^ = 0.020, *p* = 0.045). At 1 cd/m^2^ luminance level, the average pupil diameter, internal SA and corneal HOAs had an effect on the halo radius [halo = 116.877 + (25.723 × average pupil diameter) – (93.034 × internal SA) + (14.544 × corneal HOAs)] (*F* = 7.197, *p* = 0.000) with an adjusted *R*^2^ of 0.111.

## Discussion

Glare symptom assessment is often required when visual health is examined. Glare is a common concern for patients and refractive surgeons. In this study, the disk halo size of patients with myopia and myopic astigmatism was evaluated using a vision monitor device (MonPack One, Metrovision, France), and the factors potentially associated with disk halo size were analyzed. The results showed no differences in the mean halo radius between myopic and healthy eyes at 5 cd/m^2^, nor between high and low-to-moderate myopia at 5 and 1 cd/m^2^. According to a stepwise linear regression model, the internal SA had a negative effect on the halo radius at 5 cd/m^2^; the average pupil diameter, internal SA and corneal HOAs played a large role in determining the halo radius at 1 cd/m^2^.

In this study, we evaluated the disk halo size of myopic patients within −10.0 diopters, and compared the results with the values from age-matched healthy eyes reported by Puell et al. (11). In this previous study, 147 subjects with sphere within ±3.75 diopters or cylinder within ±1.50 diopters were included and stratified into six age groups (range: 20–77 years). The mean halo size for each age group and reliability of the halo measurements were determined by the same device (Metrovision, Pérenchies, France) at 5 cd/m^2^. There was no correlation between SE and halo size. Also, no significant difference in halo size was found between the high and low-to-moderate myopia groups. The mean halo radius for low photopic luminance (82.5 ± 21.8 arc min) agreed with the normal values of healthy eyes (88.4 ± 22.1 arc min) (11), unoperated eyes (80.8 ± 26.9 arc min) (17), and even younger individuals (77.2 ± 25.0 arc min) (18). Also, we measured the mean halo size (236.7 ± 52.2 arc min) at a mesopic luminance level (1 cd/m^2^). Since all measurements were obtained with optimal correction, our results could elucidate about myopic and healthy eyes. Another previous study (12) examines the halo size for a large sample of myopic patients under a photopic condition (luminance: 5 cd/m^2^). However, the measurements were conducted with the subjects' personal spectacles (hence, no optimal correction). Their results (157.4 ± 56.7 arc min) differ from those of other studies (11, 17, 18) under the same luminance condition.

The difference in halo size from said study (12) and other studies could have resulted from several issues. First, said study investigated whether the disk halo size in myopic patients differs from the normal values of healthy eyes. Furthermore, the subjects were asked to put on their personal spectacles (12), which might have been affected by defocus or surface scratch and particles, so that everyday visual experiences could be simulated. This might have caused the halo size to be different from those that were obtained when there was a proper optical correction. In addition, evidence has shown that eyes with higher myopia might exhibit larger objective scattering index values (19), which was positively correlated with the halo radius (18). Consequently, the disk halo size in eyes with ultra-high myopia (−10.0 diopters or over) might be greater than in high myopic eyes (within −10.0 diopters). Therefore, the discrepancy may be partly attributed to the differences in SE distribution between the present and previous studies (range: −10.38 to −1.00 vs. −18.00 to −1.50 diopters) (12). Furthermore, patients showed different baseline values in pupillometry parameters from those of other study (18), thereby potentially affecting the radius of halo.

The innovation of wavefront-sensing techniques has redefined the limitations of the individual optical system by wavefront aberration, including low-order aberrations (defocus, tilt, and astigmatism) and HOAs (e.g., ocular or corneal SA, coma, trefoil, etc.). Therefore, it has enabled us to accurately describe or decompose the optical components (ocular, cornea, and internal) properties of the eye. The HOAs illustrated by corneal refractive surgery are predominantly SA and coma (20–22). In addition, the relationship between patient-reported symptoms and HOAs has been investigated in patients after refractive surgery (7, 9, 10, 23, 24). However, only very weak correlations and clinically irrelevant associations between visual symptom scores and corneal HOAs have been found (9, 10). To date, little is known about the connection between the preoperative SA and disk halo size. The present data shows a weak association between the halo size and internal SA at both low photopic and mesopic luminance levels. In the human eye, there is a compensation mechanism between corneal aberrations and lens aberrations (25). We speculate that the negative (rather than positive) correlation between halo size and internal SA can be attributed to the compensation mechanism originating from internal aberrations.

In this study, Spearman correlation analysis indicated that the halo radius correlated with pupil size (initial, maximum, minimum, and average pupil diameter) and contraction amplitude at 5 cd/m^2^. These findings differ from those of Zhao et al. (12) and Yao et al. (18), who reported that halo size was only related to minimum pupil diameter under the same condition. Although pupillary responses might not be influenced by refractive errors (26–28), differences in pupil size (initial pupil diameter, pupil maxima, pupil minima, and average pupil diameter) among the three studies could be responsible for the difference. The pupil size, as controlled by the pupillary light reflex, determines both retinal illuminance and image quality (29). Theoretically, the presence of HOAs, such as SA, can push the light eccentricity of the PSF (~0.1°) so much that retinal image quality can deteriorate as a fuction of an increasing pupil size (30). In the present study, the mean halo radius was >1°. Hence, the weak relationship between the internal SA and halo radius might be attributed to their difference in the angle domain.

As for contrast sensitivity, Spearman correlation analysis showed no correlation between the halo radius and AULCSF. This finding was unexpected. The whole curve of contrast sensitivity function (CSF) could be affected by glare if contrast sensitivity was measured under glare. This could be due to the fact that contrast sensitivity was measured without glare in this study.

Our study has some limitations. Although patients without night vision disturbances were enrolled in the study, patients with high myopia over −10 diopters were not included. In addition, due to the common daily-life under-correction, which may have led to bias, a subjective evaluation was not applied. In contrast, the retinal image can be affected by HOAs, light scatter, and diffraction. However, scatter and diffraction were not evaluated in this study; this might be a potential reason for our significant, but low correlations between the variables.

In conclusion, the disk halo size of patients with myopia and myopic astigmatism within −10 diopters and correlated factors were identified. The internal SA had a negative effect on the halo radius at 5 cd/m^2^; the average pupil diameter, internal SA and corneal HOAs played a large role in determining the halo radius at 1 cd/m^2^. Our data demonstrated that the mean halo radius was not clinically different between myopic and healthy eyes at 5 cd/m^2^ luminance level and that it was not significantly different between high and low-to-moderate myopia. These results will be useful in comparing the values of ametropic patients who complain about glare after having a refractive surgery. In future studies, disk halo size in patients after refractive surgery should be investigated.

## Data Availability Statement

The original contributions presented in the study are included in the article/Supplementary Material, further inquiries can be directed to the corresponding author/s.

## Ethics Statement

The studies involving human participants were reviewed and approved by the Eye & ENT Hospital, Fudan University Ethics Committee. The patients/participants provided their written informed consent to participate in this study.

## Author Contributions

WZ, JZ, ML, and JW: data collection. TH, ML, and JW: statistical analysis and data interpretation. WZ: preparing manuscript and figures. WZ, JZ, TH, ML, and JW: critical revision of the manuscript. XZ: study concept, design, and supervision. All authors contributed to the study revision and approved the final manuscript.

## Funding

This study was supported by the National Natural Science Foundation of China (Grant No. 81770955), Joint Research Project of New Frontier Technology in Municipal Hospitals (SHDC12018103), Shanghai Science and Technology Project (Grant No. 20410710100), Major Clinical Research Project of Shanghai Shenkang Hospital Development Center (SHDC2020CR1043B), and Project of Shanghai Xuhui District Science and Technology (2020-015).

## Conflict of Interest

The authors declare that the research was conducted in the absence of any commercial or financial relationships that could be construed as a potential conflict of interest.

## Publisher's Note

All claims expressed in this article are solely those of the authors and do not necessarily represent those of their affiliated organizations, or those of the publisher, the editors and the reviewers. Any product that may be evaluated in this article, or claim that may be made by its manufacturer, is not guaranteed or endorsed by the publisher.

## Supplementary Material

Supplementary Figure 1 Metrovision's glare test. A glare-producing light source created a veiling light, which reduced the visibility of nearby optotypes set at a luminance level of 5 cd/m^2^ (left). Unrecognized letters were treated as halo radius and calculated in arc min.

Supplementary Figure 2 Metrovision's contrast sensitivity test. The contrast sensitivity function curve is demonstrated as a graph in green color connecting the averaged estimation of contrast values measured at each spatial frequency, with normal limits displayed in dark color.

Supplementary Table 1 Dynamic pupillometry data of the participants.

Supplementary Table 2 Contrast sensitivity data of the participants.
