# Supplementary figures and images for: Evaluation of Disk Halo Size and Identification of Correlated Factors in Myopic Adults
Source: Front Med (Lausanne). 2022 Jan 28;9:743543. doi: 10.3389/fmed.2022.743543 (PMC8831374; doi:10.3389/fmed.2022.743543)

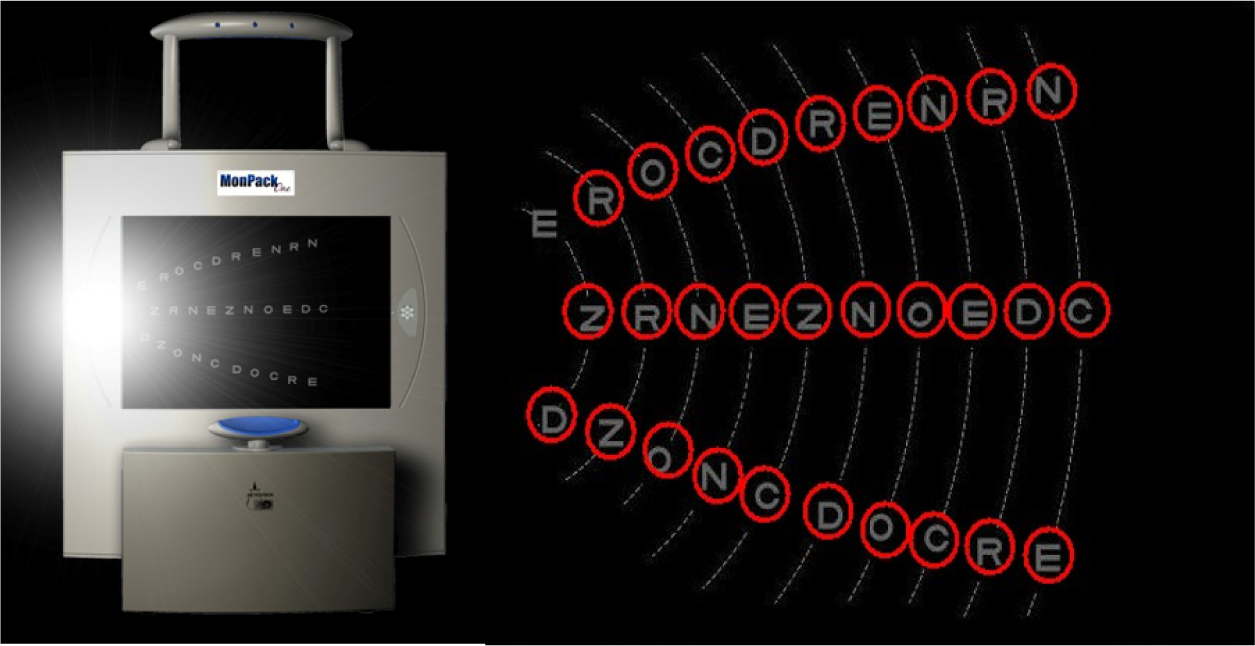

Supplement: Supplementary Figure 1 — Metrovision's glare test. A glare-producing light source created a veiling light, which reduced the visibility of nearby optotypes set at a luminance level of 5 cd/m2 (left). Unrecognized letters were treated as halo radius and calculated in arc min. [file Image_1.TIF]

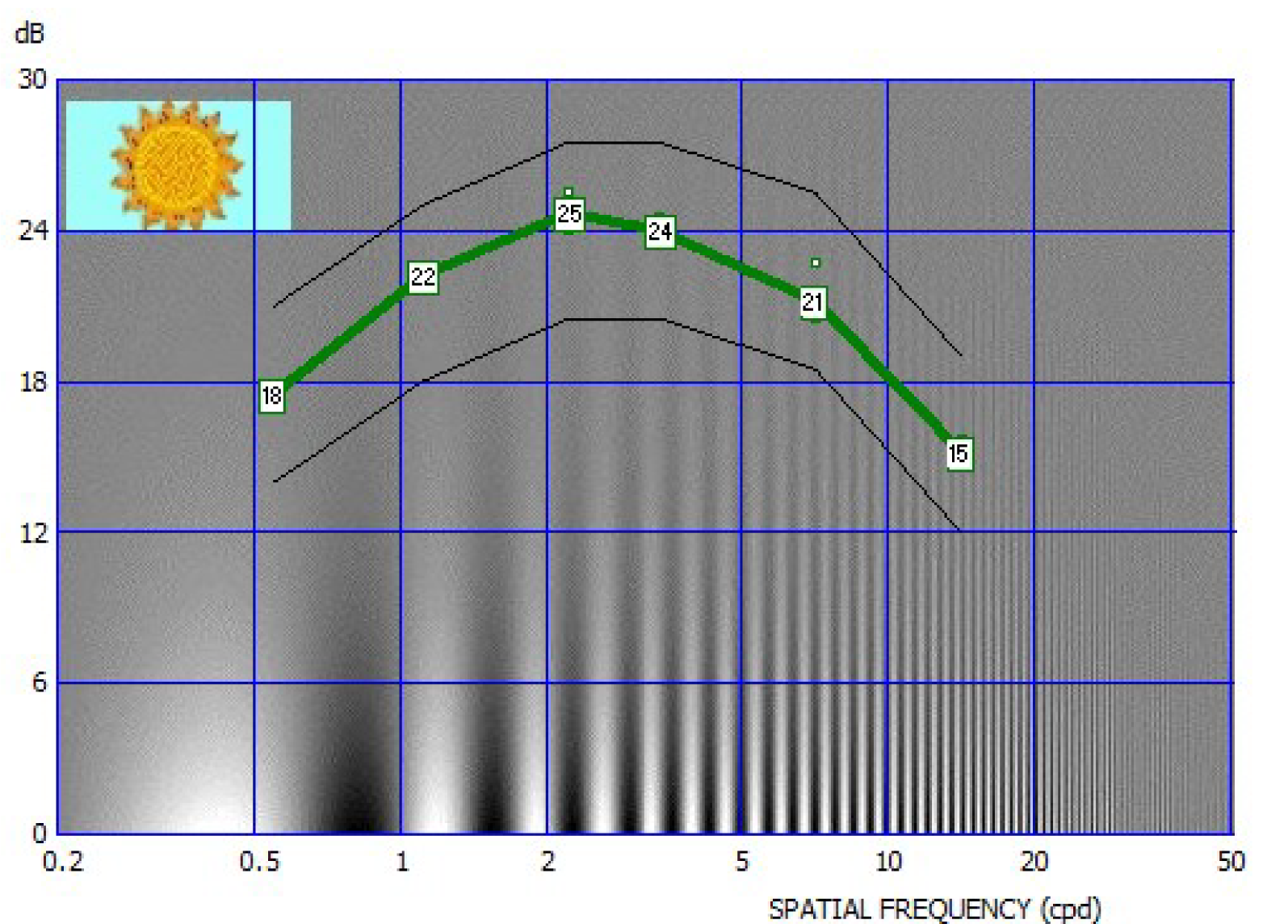

Supplement: Supplementary Figure 2 — Metrovision's contrast sensitivity test. The contrast sensitivity function curve is demonstrated as a graph in green color connecting the averaged estimation of contrast values measured at each spatial frequency, with normal limits displayed in dark color. [file Image_2.TIF]
