# Supplementary material for: Evaluation of Disk Halo Size and Identification of Correlated Factors in Myopic Adults
Source: Front Med (Lausanne). 2022 Jan 28;9:743543. doi: 10.3389/fmed.2022.743543 (PMC8831374; doi:10.3389/fmed.2022.743543)
Supplement: Supplementary Table 1 — Dynamic pupillometry data of the participants. [file Data_Sheet_1.docx]

**Supplementary Table 1** Dynamic pupillometry data of the participants (n=150)

| Dynamic pupillometry parameters | | Mean ± SD | Range |
| --- | --- | --- | --- |
| Averaged response | Initial PD, mm | 4.86±0.57 | 3.5~6.5 |
|  | Amplitude of contraction, mm | 1.82±0.24 | 1.18~2.46 |
|  | Latency of contraction, ms | 230.28±38.73 | 98~284 |
|  | Duration of contraction, ms | 622.91±57.62 | 475~784 |
|  | Velocity of contraction, mm/ms | 5.65±0.72 | 3.72~7.85 |
|  | Latency of dilation, ms | 853.19±49.46 | 720~961 |
|  | Duration of dilation, ms | 1638.46±50.95 | 1506~1748 |
|  | Velocity of dilation, mm/ms | 2.07±0.26 | 1.49~2.88 |
| Temporal response | Maximum PD, mm | 5.32±0.63 | 4.21~8.11 |
|  | Minimum PD, mm | 2.88±0.40 | 1.62~4.11 |
|  | Average PD, mm | 4.33±0.49 | 3.24~5.74 |

PD, pupil diameter.

**Supplementary Table 2** Contrast sensitivity data of the participants (n=150)

| Spatial frequencies | Mean ± SD (dB) | Range |
| --- | --- | --- |
| 0.5 cycle per degree | 16.43±2.02 | 12~22 |
| 1.1 cycles per degree | 20.42±1.74 | 15~25 |
| 2.2 cycles per degree | 21.80±1.70 | 15~26 |
| 3.4 cycles per degree | 21.57±1.88 | 15~26 |
| 7.1 cycles per degree | 19.57±2.34 | 13~26 |
| 14.6 cycles per degree | 11.86±2.34 | 6~17 |

SD, standard deviation.
